# Application of machine learning algorithms for localized syringe services program policy implementation – Florida, 2017

**DOI:** 10.1080/07853890.2022.2105391

**Published:** 2022-07-28

**Authors:** Tyler S. Bartholomew, Hansel E. Tookes, Emma C. Spencer, Daniel J. Feaster

**Affiliations:** a Division of Health Services Research and Policy, Department of Public Health Sciences, University of Miami Miller School of Medicine, Miami, FL, USA; b Department of Medicine, Division of Infectious Diseases, University of Miami Miller School of Medicine, Miami, FL, USA; c Florida Department of Health, Division of Disease Control and Health Protection, HIV/AIDS Section, Bureau of Communicable Diseases, Tallahassee, FL, USA

**Keywords:** People who inject drugs, harm reduction policy, syringe services program, policy implementation

## Abstract

**Background:**

People who inject drugs (PWID) are at an amplified vulnerability for experiencing a multitude of harms related to their substance use, including viral (e.g. HIV, Hepatitis C) and bacterial infections (e.g. endocarditis). Implementation of evidence-based interventions, such as syringe services programs (SSPs), remains imperative, particularly in locations at an increased risk of HIV outbreaks. This study aims to identify communities in Florida that are high-priority locations for SSP implementation by examining state-level data related to the substance use and overdose crises.

**Methods:**

State-level surveillance data were aggregated at the ZIP Code Tabulation Area (ZCTA) (*n* = 983) for 2017. We used confirmed cases of acute HCV infection as a proxy of injection drug use. Least Absolute Selection and Shrinkage Operator (LASSO) regression was used to develop a machine learning model to identify significant indicators of acute HCV infection and high-priority areas for SSP implementation due to their increased vulnerability to an HIV outbreak.

**Results:**

The final model retained three variables of importance: (1) the number of drug-associated skin and soft tissue infection hospitalizations, (2) the number of chronic HCV infections in people aged 18–39, and 3) the number of drug-associated endocarditis hospitalizations. High-priority SSP implementation locations were identified in both urban and rural communities outside of current Ending the HIV Epidemic counties.

**Conclusion:**

SSPs are long researched, safe, and effective evidence-based programs that offer a variety of services that reduce disease transmission and assist with combating the overdose crisis. Opportunities to increase services in needed regions across the state now exist in Florida as supported by the expansion of the Infectious Disease Elimination Act of 2019. This study provides details where potential areas of concern may be and highlights regions where future evidence-based harm reduction programs, such as SSPs, would be useful to reduce opioid overdoses and disease transmission among PWID.

**Key messages**
- The rate of acute HCV in Florida in 2017 was 1.9 per 100,000, nearly twice the national average.
- Serious injection related infections among PWID are significant indicators of acute HCV infection.
- High-priority SSP implementation locations in Florida were identified in both urban and rural communities, including those outside of current *Ending the HIV Epidemic* counties.

## 1. Introduction

Due to the convergence of the opioid and stimulant crises in the United States [1–3], there has been a significant increase in the prevalence of people who inject drugs (PWID), as well as incidence of overdose death [4,5]. PWID are at an amplified vulnerability for experiencing a multitude of harms related to their substance use, including viral (e.g. HIV, Hepatitis C) and bacterial infections (e.g. skin and soft tissue, infective endocarditis) [6–8] and fatal overdose [9]. In 2018, approximately 10% of new HIV infections were related to injection drug use (IDU) [10], and IDU has been the primary risk factor for the rising rate of acute hepatitis C virus (HCV) infections across the U.S [11]. In addition, hospitalizations related to IDU-associated bacterial infections, such as infective endocarditis, have been significantly increasing over the last 10 years [12,13].

While the number of HIV diagnoses among PWID steadily decreased between 2010 and 2015 [14], IDU-associated HIV outbreaks linked to opioid and other concurrent substance use disorders [15–20] have contributed to a significant increase in HIV diagnoses among this vulnerable population. This concerning trend has generated local and national focus on rapid recognition of HIV outbreaks and implementation of control measures to mitigate further transmission. In 2016, the Centres for Disease Control and Prevention published a nationwide assessment of U.S. counties most vulnerable to rapid spread of IDU-associated HIV [21], utilizing county-level acute HCV infection as a proxy measure for IDU. Results from this analysis highlighted two important findings: (1) social and economic conditions are significantly related to acute HCV, and (2) the most vulnerable counties lacked sufficient harm reduction-based HIV prevention strategies for PWID, specifically syringe services programs (SSPs). Recent research has corroborated these findings and has expanded to investigate the utility of risk environment frameworks to better understand the physical, social, and economic influences at the community-level, providing robust context to drivers of drug-related harms [22].

The methodology presented in Van Handel et al. (2016) has been adopted by state health departments to understand localized context of vulnerable counties for rapid HIV spread among PWID to geographically target the implementation of HIV prevention interventions [23] and has been extended to zip code [24] and census tract [25] geographical levels. These national and state-level analyses have led to a significant increases in authorization and implementation of SSPs across the country [26]. However, SSPs have, and continue to, experience significant political opposition, which have led to closures in highly vulnerable locations (West Virginia and Indiana) [27]. In 2019, the Florida Legislature passed the Infectious Disease Elimination Act (IDEA) authorizing the expansion of SSPs across the state by allowing counties to pass ordinances to implement these harm reduction programs in their respective jurisdictions [28]. Florida has been severely impacted by the syndemic of overdose, HIV infection, and HCV infection. In 2020, 6,089 Floridians died from a opioid-related overdose [29], and 7 of the 67 counties (Miami-Dade, Broward, Palm Beach, Hillsborough, Pinellas, Orange, and Duval) have been identified as high-priority counties under the *Ending the HIV Epidemic: Plan for America* initiative [30]. Taken together, with the expansion of SSPs, it is imperative to understand the highest-priority counties and zip-code level locations in Florida for local HIV prevention policy and program implementation for PWID.

The current methodology used for identifying vulnerable locations has been comprised of a multi-step process, including assessment for multicollinearity, variable reduction (e.g. principal component analysis), and regression modelling that is subject to overfitting [21,23,31]. The use of machine learning (ML) algorithms has become more common in the field of HIV prevention research, offering a flexible method to evaluate large and complex data. ML is broadly defined as the process by which computational and statistical algorithms “learn” from data [32]. There are a myriad of ML algorithms used in practice, ranging in complexity, applicability, and functionality (e.g. regression, regularization, decision tree, Bayesian, deep learning, and clustering). These learning algorithms have been applied in HIV research, including the creation of prediction tools for providers to identify candidates for PrEP [33,34], determining factors associated with HIV testing among high-risk groups [35], identifying individuals at high-risk for HIV acquisition [36], and has been recently used in predicting vulnerable locations for overdose, HIV, and HCV [25]. This study aims to identify jurisdictions in Florida that are high-priority locations for rapid SSP implementation by applying a ML algorithm to state-level data that are related to the substance use and infectious disease epidemics.

## 2. Methods

### 2.1. Study design and setting

Following the methodology used in Van Handel et al., 2016, Rickles et al., 2018, and Bergo et al., 2021, we used an ecological study design for the entire state of Florida. Counts and percentages of membership in socioeconomic features were collected from the American Communities Survey (ACS) 2013–2017 5-year estimates at the ZIP code Tabulation Area (ZCTA) level. In addition, Florida state-specific variables were collected and aggregated from multiple state government surveillance systems for 2017 at the ZIP code level (*n* = 983).

#### 2.1.1. Data

##### 2.1.1.1. Ethics statement

This study was reviewed by the University of Miami Institutional Review Board (IRB) and was declared exempt from IRB review due to the use of deidentified surveillance data in aggregate and publicly available data sources.

##### 2.1.1.2. Outcome variable

The primary outcome of interest for the present analysis was ZCTA-level incidence of acute HCV infection in the state of Florida in 2017. Data were provided by the Florida Department of Health Merlin surveillance system, Florida’s repository of clinical and laboratory data for reportable diseases [37]. Acute HCV incidence was defined as newly diagnosed by positive HCV antibody and/or positive RNA nucleic acid amplification test with discrete onset of symptoms consistent with acute viral hepatitis (e.g. fever, headache, malaise, anorexia, nausea, vomiting, diarrhea, and abdominal discomfort) and either jaundice or elevated liver enzymes (serum alanine aminotransferase [ALT] level >200 IU/L) during the period of acute illness.

##### 2.1.1.3. State surveillance variables

Twenty-six ZCTA-level features were collected across 5 Florida state surveillance systems (Table 1). Variables were selected based on the findings from Van Handel et al., 2016, Rickles et al., 2017, and theoretical indicators of acute HCV infection hypothesized by the study team. All data were aggregated as counts by ZIP code for 2017. State-level health variables included in the models were: number and rate of deaths related to all drugs, number and rate of deaths related to heroin and opioids only, number and rate of deaths related to multiple substances, rate of nonfatal drug overdoses (all drugs), rate of nonfatal drug overdoses (opioid), number and rate of sexually transmitted infections (STIs i.e. syphilis, gonorrhoea, and chlamydia), number and rate of chronic HCV infections in people between the ages of 18–39 (defined as laboratory confirmed positive HCV RNA AND does not meet the case definition of acute hepatitis C), and the number and rate of serious injection related infection (SIRI) hospitalizations. SIRI included infective endocarditis, skin and soft tissue infections (SSTIs), osteomyelitis, and bacteraemia and sepsis. SIRI were determined based on a validated ICD-10 algorithm, and more in-depth description of the methodology identifying these infections is published elsewhere [38].

**Table 1.** Florida state-specific estimates, Data Sources, and Descriptive Statistics Aggregated at the ZIP Code Tabulation Area (ZCTA) (*N* = 983).

Feature	Description	Data Source	Mean	Median	IQR
Deaths related to all drugs	The number of all deaths attributed to any kind of drug (based on residential ZCTA)	Florida Department of Child and Families	4.5	3.0	1.0–6.0
Rate of deaths related to all drugs	The number of all deaths attributed to any kind of drug per 100,000	Florida Department of Child and Families	25.9	16.1	5.7–29.4
Deaths related to heroin and opioids only	The number of all deaths attributed to heroin or opioids (based on residential ZCTA)	Florida Department of Child and Families	3.6	2.0	0–5.0
Rate of deaths related to heroin and opioids only	The count of all deaths attributed to heroin or opioids per 100,000	Florida Department of Child and Families	18.6	11.4	0–23.7
Deaths related to multiple substances	The count of all deaths attributed to multiple substances (based on residential ZCTA)	Florida Department of Child and Families	2.6	1.0	0–4.0
Rate of deaths related to multiple substances	The count of all deaths attributed to polysubstance use per 100,000	Florida Department of Child and Families	13.7	7.3	0–17.4
Rate of nonfatal drug overdoses, all drugs	Number of EMS calls for nonfatal overdose for all drugs per 100,000	Florida Drug Overdose Surveillance and Epidemiology (FL-DOSE)	43.0	20.0	4.0–50.5
Rate of nonfatal drug overdoses, heroin only	Number of EMS calls for nonfatal overdose for heroin or opioids per 100,000	Florida Drug Overdose Surveillance and Epidemiology (FL-DOSE)	16.1	3.0	0–14.0
Number of Syphilis infections	The number of syphilis infections reported to FDOH by ZCTA	MERLIN	9.1	3.0	1.0–10.0
Rate of Syphilis infections	The number of syphilis cases per 100,000	MERLIN	42.9	18.6	5.4–44.2
Number of Gonorrhoea infections	The number of Gonorrhoea infections reported to FDOH by ZCTA	MERLIN	31.6	16.0	4.0–37.0
Rate of Gonorrhoea infections	The number of Gonorrhoea cases per 100,000	MERLIN	157.9	90.3	48.1–169.1
Number of Chlamydia infections	The number of chlamydia infections reported to FDOH by ZCTA	MERLIN	101.7	65.0	23.0–134.0
Rate of Chlamydia infections	The number of chlamydia cases per 100,000	MERLIN	507.5	361.4	247.7–560.6
Acute HCV infection	The number of acute HCV infections reported to FDOH by ZCTA	MERLIN	0.41	0	0-1.0
Rate of acute HCV infection	The rate of acute HCV infection by ZCTA	MERLIN	1.9	0	0-2.4
Chronic HCV infection in persons between 18 and 39 years old	The number of chronic HCV infections reported to FDOH in persons between the ages of 18–39 by ZCTA	MERLIN	10.4	6.0	2.0-13.0
Rate of chronic HCV infection between 18 and 39-year-old	The rate of chronic HCV infection between the ages of 18–39 per 100,000 by ZCTA	MERLIN	89.4	34.7	14.0-63.6
Drug-associated infective endocarditis	The number of drug-related endocarditis hospitalizations by ZCTA	Agency for Health Care Administration (ACHA)	1.46	0	0-2.0
Rate of drug-associated infective endocarditis	The rate of endocarditis per 100,000	Agency for Health Care Administration (ACHA)	8.7	1.6	0-10.2
Drug-associated skin and soft tissue infections (SSTI)	The number of drug-related SSTI hospitalizations by ZCTA	Agency for Health Care Administration (ACHA)	7.6	4.5	1.0-11.0
Rate of drug-associated SSTIs	The rate of SSTIs per 100,000	Agency for Health Care Administration (ACHA)	57.7	27.2	9.2-50.2
Drug-associated osteomyelitis	The number of drug-related osteomyelitis hospitalizations by ZCTA	Agency for Health Care Administration (ACHA)	2.3	1.0	0-3.0
Rate of drug-associated osteomyelitis	The rate of osteomyelitis per 100,000	Agency for Health Care Administration (ACHA)	12.6	5.7	0-16.6
Drug-associated bacteraemia and sepsis	The number of drug-related bacteraemia and sepsis hospitalizations by ZCTA	Agency for Health Care Administration (ACHA)	8.6	6.0	2.0-13.0
Rate of drug-associated bacteraemia and sepsis	The rate of bacteraemia and sepsis per 100,000	Agency for Health Care Administration (ACHA)	49.9	33.2	13.7-61.0

Since state-level data were collected at the ZIP code level and ACS variables were collected at the 5-digit ZCTA-level, ZIP codes were transformed into corresponding ZCTAs using the Uniform Data System Mapper “ZIP Code to ZCTA crosswalk” calculator [39]. ZCTAs are generalized areal presentations of ZIP Code service areas created by the U.S. Census Bureau to develop a geographical boundary.

##### 2.1.1.4. ACS variables

Twenty-five features were collected from the American Community Survey (ACS) 2013–2017 5-year estimates at the 5-digit ZCTA-level (Table 2). ACS-specific estimates included in the models were: estimated total population, percentage of population aged 18–24, percentage of persons without health insurance, percentage of households with a vehicle available, percentage of people with no high school diploma (≥25 years old), per capita income, percent of people living in poverty (based on Census-defined poverty levels), income inequality Gini coefficient, percentage of the total population that is non-Hispanic White, non-Hispanic Black, and Hispanic, total housing units, number of vacant housing units, percentage of vacant housing units, number of mobile homes, percentage of mobile homes, percentage of homes with no phone service, and percentage of the population never married. Variables with non-normal distributions were log-transformed, such as per capita income.

**Table 2.** American Community Survey (ACS) 2013–2017 5-year estimates, data sources, and descriptive statistics aggregated at the ZCTA (*N* = 983).

Features	Description	Data Source	Mean	Median	IQR
Total Population	The number of civilian noninstitutionalized population per ZCTA	ACS-ID DP05	20,627	17,802	7,520–30,395
Population aged 18–24	The estimated number of people aged 18–24 in a ZCTA	ACS-ID S1501	1,801	1,294	512–2,501
Percentage of Population aged 18–24	The estimated percentage of people aged 18-24 in a ZCTA	ACS-ID S1501	8.6%	7.7%	5.8–9.4%
Percentage of population never married	The percentage of the population of a ZCTA that were never married	ACS-ID S1201	29.7%	28.3%	22.4–35.8%
Percentage of population that is Non-Hispanic White	The number of persons who reported they were not Hispanic or Latino and were of white race alone divided by the estimated total ZCTA population	ACS-ID DP05	63.6%	70.0%	48.3–83.8%
Percentage of population that is Non-Hispanic Black	The number of persons who reported they were not Hispanic or Latino and were of black race alone divided by the estimated total ZCTA population	ACS-ID DP05	13.4%	8.0%	2.8%-16.7%
Percentage of population that is Hispanic	The number of persons who reported they were Hispanic or Latino divided by the estimated total ZCTA population	ACS-ID DP05	18.6%	11.3%	5.8–24.0%
Uninsured	The number of persons without health insurance coverage per ZCTA	ACS-ID S2701	3,034	2,036	829–4,217
Percentage uninsured	The number of persons without health insurance coverage divided by total civilian population per ZCTA	ACS-ID S2701	14.3%	13.6%	9.8–17.9%
Percentage with any vehicle access	The number of households with a vehicle available divided by the total estimated number of households per ZCTA	ACS-ID B08141	39.6%	40.9%	34.5–46.3%
Percentage with no high school diplomas	The number of persons age*d* ≥ 25 years with less than a 12th grade education (including individuals with 12 grades but no diploma) divided by the estimated ZCTA population aged ≥ 25 years.	ACS-ID S1501	13.0%	11.3%	6.6–17.6%
Per capita income, (log)	The log mean income per person in the county; derived by dividing the total income of all people aged ≥ 15 years by the total ZCTA population	ACS-ID B19301	10.2	10.2	9.9–10.4
Per capita income	The mean income per person in the county; derived by dividing the total income of all people aged ≥ 15 years by the total ZCTA population	ACS-ID B19301	$29,324	$25,693	$20,761–$33,709
Gini Coefficient	Summary measure of income inequality. Values range from 0 to 1, with higher scores indicating greater inequality	ACS-ID B19083	0.44	0.44	0.41–0.48
Percentage living in poverty	Poverty levels were defined by the Census Bureau, which uses a set of money income thresholds that vary by family size and composition to determine who is in poverty. If a family’s total income is less than the family’s threshold, then that family and every individual in it is considered in poverty. The number of persons in poverty was divided by the estimated total ZCTA population	ACS-ID B17003	9.5%	8.4%	5.9–11.6%
Total housing units	The total number of housing units per ZCTA	ACS-ID DP04	9,419	8,983	3,530–14,145
Occupied housing units	The number of occupied housing per ZCTA	ACS-ID DP04	7,640	7,180	2,758–11,316
Vacant housing units	The number of vacant housing per ZCTA	ACS-ID DP04	1,779	1,253	545–2,347
Number of mobile homes	The number of mobile homes per ZCTA	ACS-ID DP04	853	424	68–1,202
Percentage of mobile homes	The total number of mobile homes by the total number of housing units.	ACS-ID DP04	14.5%	6.8%	0.8–23.7%
Percentage of vacant housing units	The total number of vacant housing units by the total number of housing units.	ACS-ID DP04	20.9%	16.7%	11.8–24.7%
Percentage of occupied housing units	The total number of occupied housing units by the total number of housing units.	ACS-ID DP04	79.1%	83.3%	75.3–88.2%
Percentage of homes with no phone service	The average percentage of the total housing units that did not have phone service	ACS-ID DP04	1.02%	0.9%	0.55–1.3%

#### 2.1.2. Statistical analysis

##### 2.1.2.1. Spatial autocorrelation

Due to the geographical nature of this analysis, we examined the spatial distribution of our outcome variable (rate of acute HCV infection) across ZCTAs to understand the spatial autocorrelation in the outcome variable. We used the global Moran’s I statistic to evaluate whether there was a significant clustering pattern in our outcome variable [40,41]. Once Moran’s I was computed, we used Monte Carlo simulation to determine the normal distribution of Moran’s I with our outcome variable if data were spatially random [42]. Upon investigation, we determined that there was significant spatial autocorrelation (Moran’s *I* = 0.0965, *p* = .001) across ZCTAs, suggesting that neighbouring ZCTAs have similar rates of acute HCV infection, with high-high and low-low clustering. To account for the significant correlation in our outcome variable, we included a spatial autocorrelation measure in our model by averaging the rate of acute HCV infection among each ZCTA’s five closest neighbours [43].

##### 2.1.2.2. Model development

**Figure 1.**
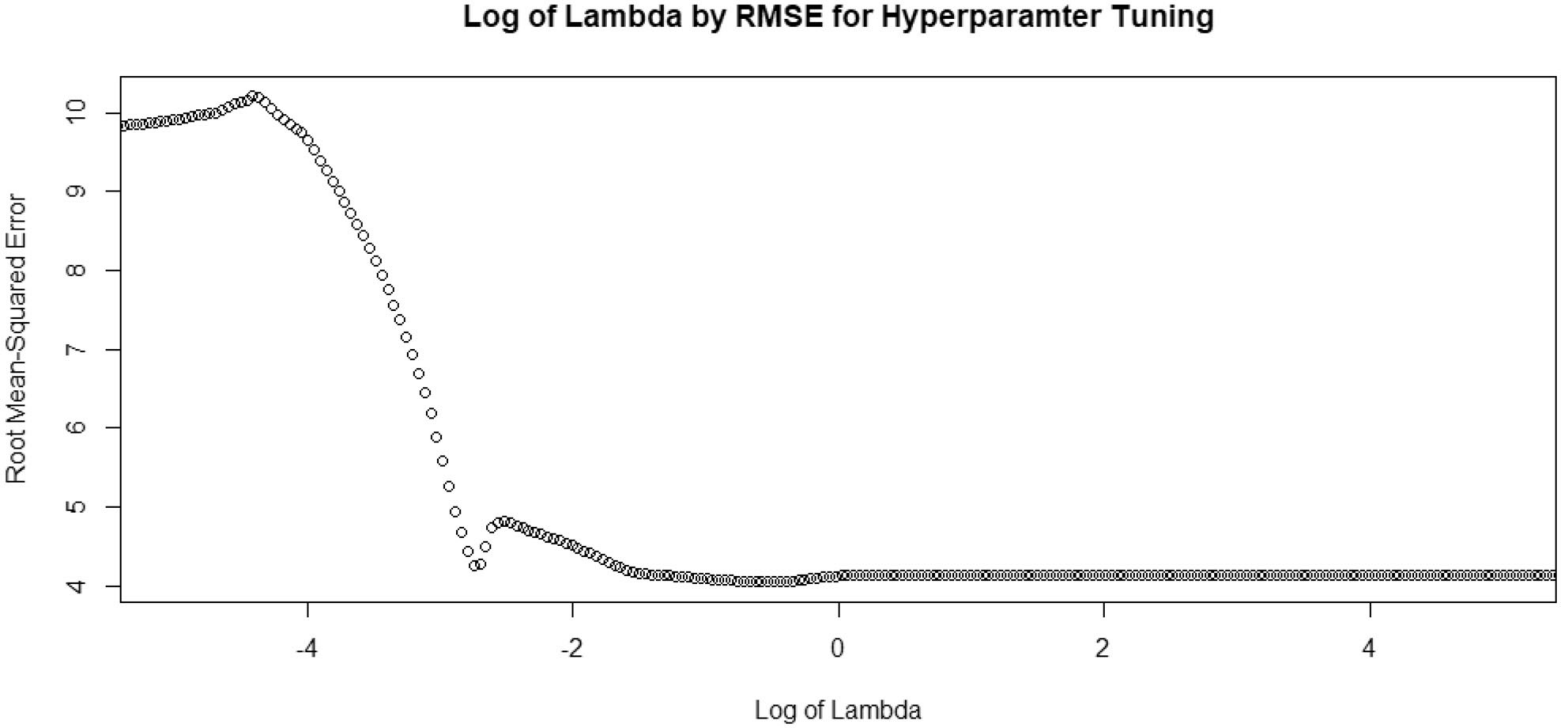
Scatterplot of root mean squared error (RMSE) across log lambda values used for training dataset. Log: logarithm; RMSE: root mean-squared error; Lambda: hyperparameter.

Using data collected from the ACS 2013–2017 5-year estimates and state-level surveillance in the state of Florida, we fitted models to predict acute HCV infection at the ZCTA-geographical level. Based on the distribution of the outcome variable, we used a standard Poisson regression model using Least Absolute Shrinkage and Selection Operator (LASSO). The LASSO regression procedure performs L1 regularization, optimizing predictive accuracy by automating the selection of variables through shrinkage and elimination of non-significant variables by setting them to zero [44]. LASSO works by applying a shrinkage penalty lambda (*λ*), or tuning hyperparameter, to the regression coefficients through minimization of the sum of squares. Increasing the lambda value increases bias in the model and allows for more and more coefficients to be set to zero and eliminated from the model (i.e. variable selection). To reduce overfitting, improve model performance, and determine the optimal regularization parameter, we divided the overall dataset into a training dataset and a validation dataset. Data were randomly split with 70% of the data being used for model training and 30% of the data being used for validating model performance. Using the training dataset only, we used k-fold cross-validation to determine the optimal, user-defined lambda value [45,46]. A vector of potential lambda values ranging from 10^−5^ to 10^5^ was created to determine optimal lambda value. The optimal lambda value was determined by the minimization of the root mean squared error (RMSE), and the optimal lambda value was used in the final model (Figure 1). Parameter estimates were determined for the model using the optimal lambda value with a Poisson distribution. Based on the number of zeros in the outcome variable (72%), we tried to fit models with a negative binomial and zero-inflated Poisson distribution. However, these models failed to converge. In addition, we explored using Random Forest (RF) as an additional specification check to assess issues with the data imbalance (preponderance of zero values) and potential high-order interactions. Due to the RF model corroborating our findings from the LASSO model, we decided to proceed with the LASSO model.

To assess how well the model performed on unseen data, the model trained on the training dataset was used to determine predictive accuracy on the validation dataset (i.e. the remaining 30% of the data). The RMSE of the model on the training and validation datasets were computed and compared for performance.

##### 2.1.2.3. Variable of importance

We further evaluated the variable importance rankings to identify which variables had the strongest predictive value of acute HCV infection. The variables selected by the model with the optimum lambda value were determined and reported to understand which variables have the most predictive power. All analysis was completed using the *caret* and *glmnet* packages in R 4.0.1 statistical environment.

##### 2.1.2.4. Vulnerability mapping

Shapefiles for 2017 ZCTAs for the state of Florida were downloaded using the *tigris* package. Shapefiles were merged with the predicted values of acute HCV infections from the training and final predictive model and mapped using the *ggplot2* package. Predicted values were split into deciles to understand the highest-priority areas for SSP implementation, defined as the 90th percentile of all ZCTAs with the highest predicted acute HCV infection. All mapping procedures were performed in R 4.0.1 statistical environment. The optimal model from the training data was used to predict outcome for both training and validation data to provide vulnerability mapping for all ZCTAs in Florida.

## 3. Results

**Figure 2.**
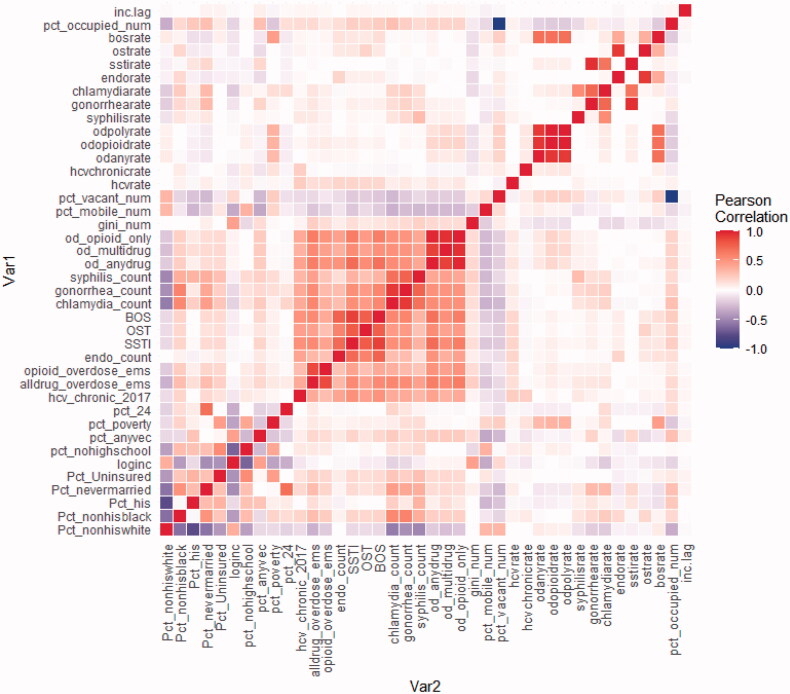
Correlation heatmap of features included in the LASSO model. *Legend*. inc.lag: Average acute HCV of neighbouring ZCTA; pct_occupied_num: percent occupied housing units; bosrate: rate of IDU-related bacteraemia and sepsis hospitalisations; ostrate: rate of osteomyelitis; sstirate: rate of skin and soft tissue infections; endorate: rate of endocarditis; chlamydiarate: rate of chlamydia; gonorrhearate: rate of gonorrhoea; syphilisrate: rate of syphilis; odpolyrate: rate of polysubstance-related overdose deaths; odopioidrate: rate of opioid-related overdose deaths; odanyrate: rate of any drug-related overdose deaths; hcvchronicrate: rate of chronic HCV among those aged 18–39; hcvrate: rate of acute HCV infection; pct_vacant_num: percent of vacant housing units; pct_mobile_num: percent of mobile homes; gini_num: GINI coefficient; od_opioid_only: number of opioid-related overdose deaths; od_multidrug: number of polysubstance-related overdose deaths; od_anydrug: number of any drug-related overdose deaths; syphilis_count: number of syphilis cases; gonorrhoea_count: number of gonorrhoea cases; chlamydia_count: number of Chlamydia cases; BOS: number of IDU-related bacteraemia and sepsis hospitalisations; OST: number of IDU-related osteomyelitis hospitalisations; SSTI: number of IDU-related skin and soft tissue infection hospitalizations; Endo_count: number of IDU-related endocarditis hospitalizations; Opioid_overdose_ems: rate of non-fatal opioid overdoses; Alldrug_overdose_ems: rate of non-fatal drug-related overdoses; Hcv_chronic_2017: number of chronic HCV infections among those aged 18–39; Pct_24: percent of population aged 18–24; Pct_poverty: percent living in poverty; Pct_anyvec: percent with any vehicle; Pct_nohighschool: percent of people with no high school education; Loginc: logarithm of per capita income; Pct_uninsured: percent of people uninsured; Pct_nevermarried: percent of people never married; Pct_his: percent of people identifying as Hispanic; Pct_nonhisblack: percent of people identifying as non-Hispanic Black; Pct_nonhiswhite: percent of people identifying as non-Hispanic White.

In 2017, of the 983 ZCTAs in Florida, 404 acute HCV infections were reported to the Florida Department of Health, with an overall incidence of 1.9 per 100,000, nearly twice the national average [47]. Acute HCV incidence across ZCTAs ranged from 0 to 46.9 per 100,000. A detailed overview of each feature’s description, data source, mean, median, and interquartile range (IQR) is presented in Tables 1 and 2 and a correlation matrix of all features is presented in Figure 2.

### 3.1. Results of the training LASSO and model validation

**Figure 3.**
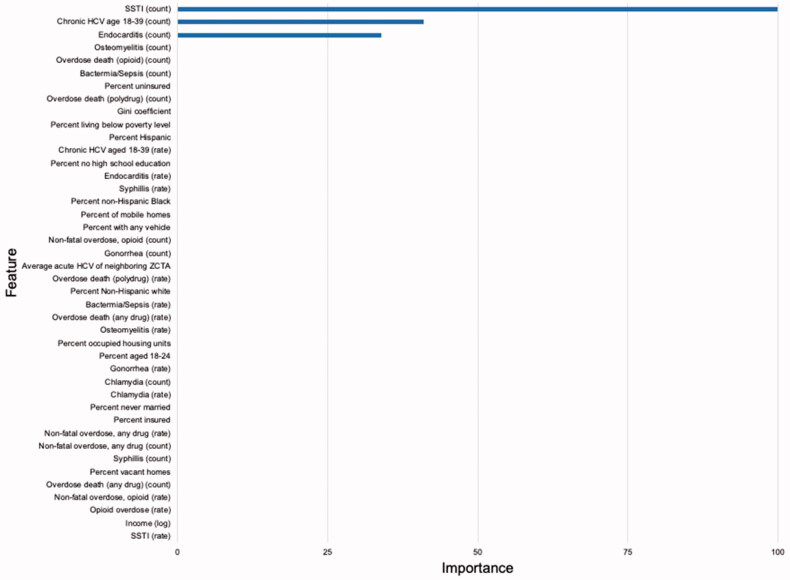
Variable Importance Index (VIMP) from the LASSO model. LASSO: least absolute shrinkage and selection operator; VIMP: variable of importance; Feature: Variable; SSTI: skin and soft tissue infections.

Using 10-fold cross-validation, the optimal lambda value in the LASSO training model that produced the lowest RMSE was *λ* = 0.561 (Figure 1). At this value, the RMSE of the model was 4.04. Of the 40 features, the LASSO variable selection procedure retained 3 predictors in the model. The strongest predictors were: (1) the number of drug-associated skin and soft tissue infection hospitalizations, (2) the number of chronic HCV infections in people aged 18–39, and (3) the number of drug-associated infective endocarditis hospitalizations (Figure 3). All other features were set to zero and eliminated from the model. When applied to the validation dataset, the RMSE of model was 4.44, suggesting the model had good predictive performance and minimal overfitting.

### 3.2. Vulnerability mapping

**Figure 4.**
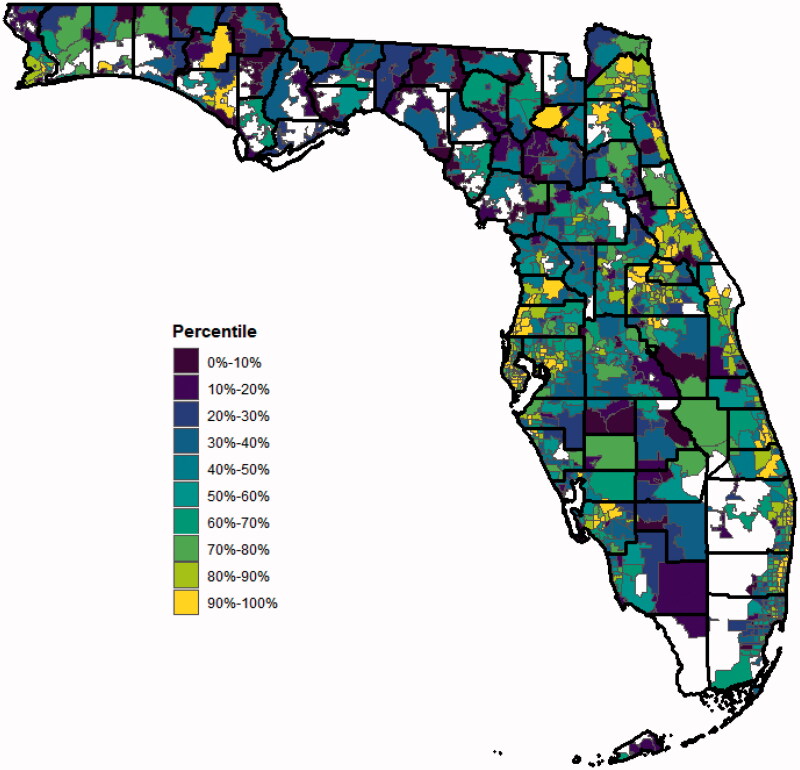
Map of predicted acute HCV percentiles by ZCTA produced by the LASSO model. Solid black lines indicate county lines; solid gray lines indicate ZCTA boundary; white space represents water or protected land (e.g. Everglades).

Based on the predicted values obtained from the training and validation model, high-priority areas were located both in urban and rural settings, even outside of the current *Ending the HIV Epidemic* jurisdictions (Figure 4). There were 27 counties that contained the 99 ZCTAs that were identified as high priority (Table 3). Counties that contained high-priority ZCTAs include (ordered from most to least): Pinellas, Duval, Palm Beach, Pasco, Broward, Orange, Volusia, Lee, Hillsborough, St. Lucie, Hernando, Bay, Brevard, Clay, Manatee, Miami-Dade, Sarasota, Seminole, Charlotte, Escambia, Martin, Okaloosa, Osceola, St. Johns, Sumter, Union, Washington. Of these counties, 5 (17.9%) have implemented SSPs, 1 (3.6%) has passed a local ordinance authorizing an SSP but have not moved to implement, and 22 (78.6%) have no SSP ordinance in place.

**Table 3.** Descriptive statistics of Florida counties containing high-priority ZCTAs*.

County	Number of ZCTAs identified as high priority
Pinellas	13
Duval	8
Palm Beach	8
Pasco	8
Broward	7
Orange	7
Volusia	6
Lee	6
Hillsborough	5
St. Lucie	4
Hernando	3
Bay	2
Brevard	2
Clay	2
Manatee	2
Miami-Dade	2
Sarasota	2
Seminole	2
Osceola	2
Charlotte	1
Escambia	1
Martin	1
Okaloosa	1
St. Johns	1
Sumter	1
Union	1
Washington	1
Total	99

*High priority jurisdictions were defined as the 90th percentile of all ZCTAs with the highest predicted rate of acute HCV infection.

## 4. Discussion

This ecological study provides important information regarding high-priority locations in Florida for the implementation of HIV prevention programs (i.e. SSPs) to serve PWID, a population vulnerable to the rapid transmission of HIV infection [15,17,18,48]. Our analysis provides state, county, and community-level stakeholders (e.g. health departments) granular information regarding where resource allocation should be focused and planning for localized SSP implementation. This study also highlights the utility of state-level surveillance data integration across departments and data sources. Through the application of a machine learning algorithm, we identified significant indicators for acute HCV infection, such as chronic HCV infection among people aged 18–39, drug-associated skin and soft tissue hospitalizations and drug-associated infective endocarditis hospitalizations.

Our data suggest a significant relationship between chronic HCV among people aged 18–39 and acute HCV incidence. Previous research has suggested that there is a plausible mechanistic relationship between chronic HCV and HCV incidence through geographical variability in community viral load [49]. Areas with high burden of active and untreated HCV may serve as a HCV reservoir, increasing the probability of HCV being transmitted during sharing of injection equipment among PWID in the absence of prevention [50]. With increasing prevalence of younger PWID [51] and increasing rates of chronic HCV among persons under the age of 39 years old [11] coupled with limited access to curative HCV treatment due to sobriety restrictions and a historical lack of HCV prevention (i.e. SSPs) among PWID in Florida, a multifaceted approach through treatment access and scaling up prevention remains imperative in the control of HCV.

These results also expand on state-level variables collected in existing surveillance systems by examining IDU-associated bacterial infections among a cohort of PWID identified by ICD-10 codes. The results from the final model highlight the compounding harms that PWID face outside of viral infections (e.g. HCV and HIV), suggesting that a state-wide surveillance system of bacterial infections (e.g. infective endocarditis) should be developed to better track and understand the trends of infectious sequelae due to the substance use and overdose crises.

The machine learning algorithm predicted well but showed room for improvement in prediction performance with the algorithm’s RMSE value >4 and R-squared value <0.10. RMSE is an absolute measure of fit, providing information on how close the observed data points are to the model’s predicted values [52]. This may be, in part, due to the relative imbalance in acute HCV infections. Many (72%) of the ZCTAs did not report any acute HCV infection, and modelling of relatively rare events can be difficult. Because LASSO regression simultaneously performs variable selection/retention, we produced a parsimonious model of 3 features which improves simplicity in understanding the final model.

This analysis contextualizes, geographically, high-priority ZCTAs for implementation of prevention services for PWID (Figure 4). With the expansion legislation passed to allow all counties in Florida to implement SSPs in 2019, counties that contain ZCTAs in the 90^th^ percentile should emergently look to support and pass local legislation to implement these evidence-based programs. The effectiveness and cost-effectiveness of SSPs as a public health strategy are well established [53–56], garnering support from the Centres for Disease Control and Prevention and explicitly named as a cornerstone program in the “Prevent” pillar of the *Ending the HIV Epidemic* initiative. To date, there have been 9 counties (Miami-Dade, Broward, Palm Beach, Hillsborough, Pinellas, Manatee, Leon, Alachua and Orange) that have passed local ordinances authorizing an SSP within their respective jurisdictions, 7 of which were identified as counties containing high-priority ZCTAs. While the majority of high-priority counties under the *Ending the HIV Epidemic* initiative have passed ordinances, this analysis highlights additional locations where local SSP implementation is imperative, including both urban (85%) and rural (15%) counties (defined by the 2010 Census). The counties identified in this analysis closely match the drug-related overdose deaths by county in 2017 [57], highlighting the syndemic opioid and overdose crises faced by Florida counties.

Based on the significant predictors of acute HCV infection, state policymakers and community stakeholders should assess the implementation of harm reduction and behavioural interventions in medical-based settings, such as emergency departments where PWID are frequent utilizers [58]. There has been increased focus on the integration of addiction medicine and infectious disease specialties to develop “Serious Injection-Related Injury (SIRI)” teams due to the significant increase in infections like infective endocarditis [59,60]. These teams are focused on providing both gold standard antibiotic therapies and evidence-based substance use disorder treatment among patients hospitalized with SIRIs [61,62] to optimize health-related outcomes. These teams are well positioned to deliver harm reduction interventions to PWID, including linkage to HIV prevention (e.g. PrEP), HIV and HCV treatment, and outpatient medications for opioid use disorder [63].

Beyond additional interventions, these findings, and the model, have important implications for the prediction and prevention of IDU-associated HIV outbreaks. Research has demonstrated that outbreaks of IDU-associated HCV may proceed the rapid transmission of HIV, most salient in the Scott County, Indiana outbreak [64,65]. In 2018, Miami-Dade county detected an outbreak of HIV among their PWID population after the implementation of an SSP in December 2016 [15]. Based on the results of this model using data from 2017, Miami-Dade county contained 2 ZCTAs that were identified as high-priority areas, of which one was the exact ZCTA where the outbreak was identified, investigated, and mitigated by the local SSP and the Florida Department of Health. This convergence of predicted and detected outbreaks may highlight the practical utility of this model to identify outbreaks in Florida. In addition, bacterial infections, such as SSTIs and infective endocarditis, could be further upstream indicators of HCV and HIV infection, highlighting the importance of incorporating these infections in the prediction of HIV outbreaks in future research [66].

### 4.1. Limitations

This analysis is subject to several limitations. First, there is a lack of accurate and robust surveillance reporting for acute HCV infection and other state-level data, such as drug-related deaths and EMS calls for a drug-related overdose. This also includes changes in case definitions over time, underreporting, and misclassification that can cause issues with the reliability of the data being modelled. However, we utilized only 2017 data on acute HCV infection in which a consistent case definition was applied across the year, and these data are the best measures available at the state level. In addition, PWID often avoid health care services due to pervasive stigma [67] remain hesitant to call 911 when responding to an overdose [68], and use naloxone distributed by SSPs in the field [69] suggesting that existing data sources are limited in capturing representative metrics. However, at the time of this study, Miami-Dade was the only county with street-level distribution of naloxone so these unreported nonfatal overdoses would not impact the model outside of Miami-Dade County in 2017. Second, our data were only limited to a cross-sectional framework, not allowing for forecasting and including spatiotemporal dynamics in the data to map risk in space and time. In addition, the final model from our 10-fold cross-validation was used to make predictions on both the training and validation datasets in order to obtain predicted values for all ZCTAs for vulnerability mapping, therefore the values for the training data are fitted values and the values for the validation data are truly predicted values. Therefore, the two subsets of ZCTAs may have differing accuracy. Third, the most significant variables in our models were variables that are not routinely collected by the state. This exclusion poses potential issues with the ability to rapidly apply this methodology to new data when available, although it does point to potential important data to add to the state’s surveillance efforts. The Agency for Health Care Administration (AHCA) in Florida is responsible for collection and management of claims data which could be utilized to provide these data on a timely basis. Fourth, this study utilized “black-box” prediction algorithms that increase the complexity of understanding how and which variables are driving prediction. However, Variable Importance Index (VIMP) can provide insights into how variables influence prediction by ranking which variables are most important in the model. Fifth, the machine learning algorithm used can be sensitive to class imbalance, which may have resulted in suboptimal predictive performance of the model. Zero-inflated, negative binomial, and Random Forest models were explored; however, the zero-inflated and negative binomial models did not converge and the Random Forest model corroborated our results from the LASSO model. Lastly, high correlation between features in the models may have impeded model performance and variable importance (Figure 2). Nonetheless, taken together, this analysis provides a more robust methodology and granular understanding of high-priority areas for SSP implementation.

## 5. Conclusions

SSPs offer a multitude of benefits for PWID. This study provides an application of machine learning algorithms that can help provide a streamlined methodology to be used by states undertaking their own vulnerability assessments. Future research should explore longitudinal modelling approaches in order to improve prediction and forecasting of risk in space and time. This study also expands on the geographical unit of analysis, providing granular data at the ZCTA-level instead of the county-level. The results from this analysis should be disseminated to local health departments to inform the targeted expansion of services for PWID, including SSPs, HIV/HCV testing and treatment, naloxone distribution, and community outreach to prevent HCV and HIV infection among this high incidence community.

## Data Availability

Data from the American Communities Survey are publicly available. Data from the Florida Department of Health are not publicly available but may be accessed upon request.
